# Reply: Early-onset Behr syndrome due to compound heterozygous mutations in *OPA1*

**DOI:** 10.1093/brain/awu187

**Published:** 2014-07-10

**Authors:** Patrick Yu-Wai-Man, Patrick F. Chinnery

**Affiliations:** 1 Departments of Neurology and Ophthalmology, Royal Victoria Infirmary, Newcastle upon Tyne, UK; 2 Wellcome Trust Centre for Mitochondrial Research, Institute of Genetic Medicine, Newcastle University, Newcastle upon Tyne, UK

Sir, Autosomal dominant optic atrophy (DOA) is the most commonly diagnosed inherited optic neuropathy in clinical practice and the majority of patients harbour pathogenic mutations within the *OPA1* gene (3q28-q29, OMIM 165500) (Yu-Wai-Man and Chinnery, 2013). OPA1 is a multifunctional protein located within the mitochondrial inner membrane and it regulates a number of critical cellular functions, including mitochondrial network stability, oxidative phosphorylation and mitochondrial cell death pathways (Lenaers *et al.*, 2009). Until recently, DOA was largely viewed as a limited genetic disorder that preferentially affects retinal ganglion cells resulting in progressive visual failure from early childhood (Carelli *et al.*, 2004; Yu-Wai-Man *et al.*, 2014). It is now abundantly clear that pathogenic *OPA1* mutations can have much more severe multisystemic consequences that are detrimental not only to optic nerve function, but also target other tissues that are frequently involved in other well-established mitochondrial syndromes (Amati-Bonneau *et al.*, 2008; Hudson *et al.*, 2008). In a large multicentre study published in *Brain*, up to 20% of *OPA1* mutation carriers developed these so-called DOA plus (DOA+) phenotypes where the optic atrophy was complicated by a wide range of neuromuscular features that included ataxia, myopathy, peripheral neuropathy, sensorineural deafness, and fascinatingly, chronic progressive external ophthalmoplegia (Yu-Wai-Man *et al.*, 2010).

A previous case report in *Brain* described two brothers diagnosed with classical Behr’s syndrome who were eventually found to carry a single heterozygous pathogenic *OPA1* mutation (c.1652G>A, p.Cys551Tyr) within the catalytic GTPase domain (Marelli *et al.*, 2011; Yu-Wai-Man and Chinnery, 2011). In their case series, Bonneau and colleagues extend the association between pathogenic *OPA1* mutations and Behr’s syndrome with a detailed account of four unrelated children who developed the typical clinical features of an early-onset progressive optic neuropathy that was further compounded by ataxia, spasticity and peripheral neuropathy (Bonneau et al., 2014). Their most striking observation is the identification of compound heterozygous *OPA1* mutations in all four patients with the co-occurrence of a missense GTPase mutation and a truncative nonsense mutation. Interestingly, three of these families harboured the same missense GTPase *OPA1* mutation (c.1146A>G, p.Ile382Met) that has been previously reported in another DOA+ family with compound heterozygous mutations (Schaaf *et al.*, 2011). This specific pathogenic variant is clearly highly penetrant for the neurological ‘plus’ features and it does support our earlier observation that misssense GTPase *OPA1* mutations seem to have a more potent deleterious impact, possibly via a dominant negative mechanism and increased mitochondrial DNA instability (Yu-Wai-Man *et al.*, 2010; Yu-Wai-Man and Chinnery, 2012). As Bonneau *et al.* (2014) correctly point out, we did describe two siblings from a non-consanguineous Norwegian family in our original *Brain* paper, who developed a particularly aggressive disease course characterized by ataxia, spasticity, peripheral neuropathy and myopathy (Yu-Wai-Man *et al.*, 2010). *OPA1* sequencing identified two pathogenic variants in both the affected brother and sister: the c.768C>G (p.Ser256Arg) missense mutation in exon 5b and the c.854A>G (p.Gln285Arg) missense mutation in exon 8. Bonneau *et al.* (2014) rightly queried whether we had actually proven compound heterozygosity in these two affected Norwegian siblings. Although DNA was not available from their deceased parents, we did have access to DNA samples from the brother’s two unaffected daughters and both harboured only the c.768C>G (p.Ser256Arg) substitution in exon 5b. Furthermore, haplotype analysis provided additional evidence that the proband and his affected sister were indeed compound heterozygous for the c.768C>G (p.Ser256Arg) and the c.854A>G (p.Gln285Arg) *OPA1* mutations (Yu-Wai-Man *et al.*, 2010).

Three *Opa1* mouse models have been developed harbouring truncative mutations in exon 8 (c.1051C>T) (Davies *et al.*, 2007), intron 10 (c.1065+5 G>A) (Alavi *et al.*, 2007), and exon 27 (c.2708–2711delTTAG) (Sarzi *et al.*, 2012). Heterozygous mutant mice exhibited ∼50% reduction in overall protein expression, in keeping with a haploinsufficiency mechanism, and these mice faithfully replicated the human phenotype with a slowly progressive bilateral optic neuropathy and reduced visual parameters. Optic nerve degeneration was documented as early as 6 months, but it was much more striking by 2 years of age. Interestingly, in all three *Opa1* mouse models, homozygous mutant mice died *in utero* during early embryogenesis, clearly highlighting the central role played by OPA1 in early development. This major profusion protein has been highly conserved throughout evolution and it is perhaps not surprising that so far, no affected individuals have been reported that carry homozygous or compound heterozygous nonsense or frameshift *OPA1* mutations, which are likely to be embryonically lethal.

The final clinically relevant point that we would like to make relates to the use of Behr’s syndrome (OMIM 210000) as a diagnostic label. In 1909, Carl Behr, a German ophthalmologist, described an infantile form of optic atrophy complicated by mental retardation and spinocerebellar degeneration that resulted in ataxia, spasticity and peripheral neuropathy (Behr, 1909). The genetic advances of the past two decades have transformed our understanding of human diseases and with the greater availability of next-generation sequencing technology, it has become apparent that most eponymous syndromes have a heterogeneous molecular genetic basis and should be viewed as largely historical descriptions. Behr’s syndrome is a very good illustration of this fundamental shift in genetic disease classification, based not solely on the clustering of recognizable clinical features, but primarily on the identification of the underlying genetic defects. This syndromic inherited optic neuropathy was originally linked to autosomal recessive *OPA3* mutations among Iraqi Jewish patients with elevated urinary excretion of 3-methylglutaconic acid and 3-methylglutaric acid—a subtype that was known by yet another eponymous description, namely Costeff syndrome (Costeff *et al.*, 1989; Anikster *et al.*, 2001). Besides *OPA3*, we now know that both single and compound heterozygous *OPA1* mutations can result in multisystemic DOA+ phenotypes that would be entirely consistent with Carl Behr’s original case report. This is certainly not the end of the story and the list of disease-causing genes is bound to grow even further, a fact that is clearly exemplified by the recent identification of compound homozygous *C12orf65* mutations in patients with phenotypic manifestations indistinguishable to those classically associated with ‘Behr’s syndrome’ (Pyle *et al.*, 2014). Downregulation of the C12orf65 protein results in a mitochondrial translation defect and profound multiple respiratory chain defects. Despite the underlying genetic heterogeneity, a unifying theme is clearly emerging in ‘Behr’s syndrome’ with mitochondrial dysfunction being the final common pathway that is ultimately leading not only to retinal ganglion cell loss and optic nerve degeneration, but also to more widespread neuronal loss with multisystemic manifestation. Generic treatment modalities aimed at correcting these dysfunctional mitochondrial mechanisms could therefore prove beneficial to this group of patients irrespective of the causative genetic defect.

## Funding

P.Y.W.M. is a Medical Research Council (MRC, UK) Clinician Scientist. P.Y.W.M. also receives funding from Fight for Sight (UK) and the UK National Institute of Health Research (NIHR) as part of the Rare Diseases Translational Research Collaboration. P.F.C. is a Wellcome Trust Senior Fellow in Clinical Science and a UK National Institute of Health Research (NIHR) Senior Investigator who also receives funding from the MRC (UK) and the UK NIHR Biomedical Research Centre for Ageing and Age-related disease award to the Newcastle upon Tyne Hospitals NHS Foundation Trust.
